# Envisioning a role for nuclear actin in prophase I spermatocytes

**DOI:** 10.3389/fcell.2023.1295452

**Published:** 2023-11-24

**Authors:** Jana Petrusová, Jasper Manning, Dominik Filipp

**Affiliations:** Laboratory of Immunobiology, Institute of Molecular Genetics of the Czech Academy of Sciences, Prague, Czechia

**Keywords:** actin, prophase I, nucleoskeleton, chromatin remodeling, spermatoproteasome

## Abstract

Actin is a multi-functional protein that is involved in numerous cellular processes including cytoskeleton regulation, cell migration, and cellular integrity. In these processes, actin’s role in respect to its structure, complex mechanical, and protein-binding properties has been studied primarily in the cytoplasmic and cellular membrane compartments. However, its role in somatic cell nuclei has recently become evident where it participates in transcription, chromatin remodeling, and DNA damage repair. What remains enigmatic is the involvement of nuclear actin in physiological processes that lead to the generation of germ cells, in general, and primary spermatocytes, in particular. Here, we will discuss the possible role and nuclear localization of actin during meiotic prophase I and its interaction with chromatin remodeling complexes, the latter being essential for the control of pairing of homologous chromosomes, cross-over formation, and recombination. It is our hope that this perspective article will extend the scope of actin’s nuclear function in germ cells undergoing meiotic division.

## 1 Introduction

Actin, a multi-functional protein, is known to be an important player in various cellular processes and has been shown to be part of cytoskeletal microfilaments and thin filaments within muscle fibrils (Henderson et al., 2017). It has also been described as an essential protein within the cytoskeleton that is necessary for cell contraction and mobility during cell division (Lappalainen, 2016). In the cytoplasm, actin forms a highly versatile and dynamic filamentous network that is involved in shaping of the cell, distribution of cellular organelles, cellular motility, and cell adhesion (Humphries et al., 2007; Svitkina, 2018). Although numerous studies have shown the presence of actin in cell nuclei, only a few have provided details of its function. However, what has been established is that nuclear actin in somatic cells is required for transcriptional processes which are initiated by all three RNA polymerases (Hofmann et al., 2004; Philimonenko et al., 2004; Percipalle, 2013), chromatin remodeling (Dundr et al., 2007; Baarlink et al., 2017), and DNA damage repair (Caridi et al., 2018; Schrank et al., 2018). It is of note, that in addition to actin, interacting partners such as actin-binding proteins (ABPs) and actin-related proteins (Arps) also reside in the nucleus (Kristo et al., 2016; Virtanen and Vartiainen, 2017).

Structurally, actin is found in one of two forms: monomeric (globular-, G-actin) or polymeric (filamentous-, F-actin), the latter forming double-stranded helical filaments, which may assemble into higher-order three-dimensional structures known as bundles (Lappalainen, 2016). In regards to the structure of actin in the nucleus, several studies have suggested that it appears either in monomeric or oligomeric form, although other studies have shown that nuclear actin also exists in a polymeric state (Oma and Harata, 2011; Kalendova et al., 2014; Plessner et al., 2015; Plessner and Grosse, 2015; Le et al., 2020; Yamazaki et al., 2020). The shift from an oligomeric to a polymeric state (and *vice versa*) is a highly dynamic process that facilitates the movement of chromosomal loci, and thus increases the chromosomal mobility of activated gene loci to transcriptionally permissive areas (Lusic et al., 2013; Baarlink et al., 2017; Plessner and Grosse, 2019) or double-strand breaks to sites where they undergo DNA damage repair (Schrank et al., 2018; Caridi et al., 2019; Schrank and Gautier, 2019). The shortening and extension of nuclear actin is also important for the positioning of chromosomes to their territories (Ondrej et al., 2008). While the positioning of these chromosomes requires a force that occurs by actin polymerization where actin becomes associated with DNA, the details of this contractile mechanism is not well understood (Hurst et al., 2019; Hyrskyluoto and Vartiainen, 2020). Importantly, others have reported that the binding of monomeric actin to DNA occurs via its engagement with chromatin remodeling complexes such as SWI/SNF (SWItch/Sucrose Non-Fermentable), INO80 (INOsitol requiring 80) and NuA4 (NuA4 histone acetyltransferase complex). This scenario directly implicates nuclear actin in the regulation of gene expression (Chuang et al., 2006; Dundr et al., 2007; Kapoor and Shen, 2014; Baarlink et al., 2017) and DNA damage repair in somatic nuclei (Belin et al., 2015a; Belin et al., 2015b; Caridi et al., 2018; Schrank et al., 2018). It is important to emphasize that chromatin remodeling, which has been primarily investigated in somatic nuclei, is also a major event in the progression of meiotic prophase I. However, there is a significant information gap in regards to the role of actin that occurs during chromatin remodeling in meiosis.

## 2 Major events of prophase I

**FIGURE 1:**
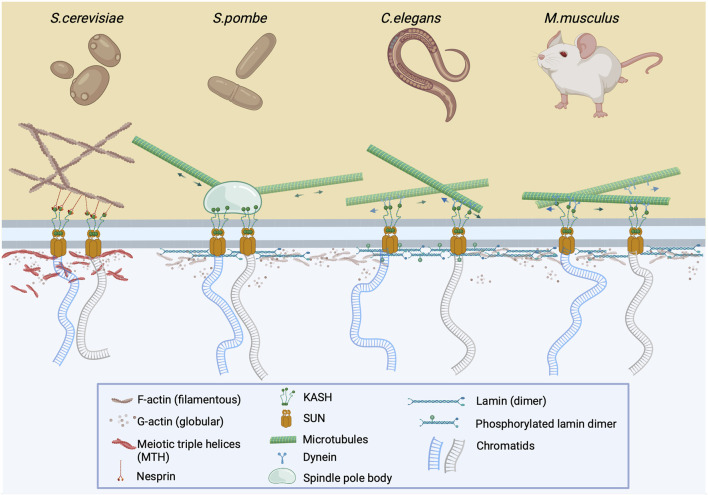
The LINC complex is instrumental for meiotic chromosome pairing and nuclear bundle formation. During early prophase I, prior to chromosome pairing, chromatids are attached to the LINC complex via SUN and KASH proteins which cross the inner and outer nuclear membrane, respectively. This complex is attached to either cytoplasmic F-actin via nesprin (*S. cerevisiae*) or to microtubules via spindle pole body (*S. pombe*), or dynein (*C. elegans* and *M. musculus*). The clustering and pairing of chromosomes at the nuclear membrane after depolymerization of cytoskeletal actin results in the formation of nuclear actin bundles or meiotic triple helices (MTHs) (Takagi et al., 2021; Ma et al., 2022). In other organisms, it is assumed that nuclear actin exists in monomeric or oligomeric form, which may be associated with phosphorylated lamin (*C.elegans*) or lamin itself (*M.musculus*) at the nuclear periphery.

During prophase I of meiosis, condensed chromosomes locate their homologous pairs, recombine, and redistribute evenly to daughter cells. This process is facilitated by the anchoring of chromosomes to the nuclear membrane via the LINC (LInker of Nucleoskeleton and Cytoskeleton) complex. This transmembrane complex is comprised of SUN and KASH proteins which interconnect chromosomes in the inner nuclear space to the cytoskeleton (see Figure 1 for details). LINC has been described as the driving force of chromosomal movement at this stage of meiosis (Trelles-Sticken et al., 2005; Chikashige et al., 2006; Chikashige et al., 2007; Koszul and Kleckner, 2009; Vogel et al., 2009; Yoshida et al., 2013; Lee et al., 2015). Meanwhile, in the nucleus, during the four stages of prophase I, *i.e.*, leptotene, zygotene, pachytene, and diplotene, processes such as DNA replication, chromatin condensation, identification of homologous DNA loci, and formation of double-strand breaks (DSBs) occur and which are subsequently followed by the recombination of non-sister chromatids (Kleckner, 1996). In spite of continuous chromosomal condensation, these events are supported by the expression of meiosis-specific genes such as SPO11 (Baudat et al., 2000), MEIOB (Xu et al., 2017), MSH4 and MSH5 (Kneitz et al., 2000; Snowden et al., 2008), which are essential for cross-over formation and meiotic recombination. Successively, replicated chromatin from the leptotene stage is paired during the zygotene stage via DNA-to-DNA interactions along with protein-to-protein stabilization (Lenormand et al., 2016). In the pachytene stage, when programmed DNA breaks are generated, fully associated and stabilized bivalents are detected and undergo recombination. This results in the generation of chiasmata, a specialized X-like chromatin configuration that develops between non-sister chromatids (Keeney and Kleckner, 1996; Kleckner, 1996; Storlazzi et al., 1996). The diplotene stage follows shortly thereafter, during which the sister chromatids segregate (Baudat et al., 2013).

It is important to emphasize that events that accompany prophase I, *i.e.*, DNA replication, chromatin condensation, DSB formation, and DNA repair are not exclusive to meiosis. These processes have been studied individually in various somatic cell types in regards to their physiological requirements in cell cycle, all of which have demonstrated a role of nuclear actin in the nucleus. However, the uniqueness of prophase I is that these processes occur successively in short sequences of highly coordinated and regulated events. Unfortunately, there has not been a conceptual study which has addressed the association of nuclear actin with these processes. To this end, we will present publicly available data which show the function of nuclear actin in somatic cells and draw some correlation to analogous processes that are associated with prophase I to create a model of actin function during meiotic events. We will specifically review the involvement of actin in prophase I of budding (S. *cerevisiae*) and fission yeast (*S. pombe*), nematode (*C. elegans)*, and mouse (*M. musculus*). To avoid repetition of published data, we will omit from this discussion the process of oogenesis which has been previously reviewed (Uraji et al., 2018; Mogessie, 2019).

## 3 Actin in prophase I nuclei

As previously noted, the movement of meiotic chromosomes that are embedded in the nuclear membrane is controlled by the LINC complex which is attached to the cytoskeleton. However, it appears that this “chromosome dance” is also controlled by the nucleoskeleton which contains nuclear actin. Trelles-Sticken et al., 2005 described the clustering and pairing of chromosomes at the nuclear membrane even after depolymerization of the cytoskeletal actin in budding yeast, a finding that changed the perception of actin as an exclusive cytoplasmic factor of prophase I progression. As a result, the authors proposed that nuclear actin perpetuated the gathering of motile telomeres by compressing chromosome ends into a limited nuclear region. Nearly16 years later, Takagi et al., 2021 confirmed this clustering by detecting bundles which contained actin in prophase I nuclei of budding yeast, which were referred to as nuclear bundles. Interestingly, Ma *et al.* observed that these nuclear actin bundles consisted of multiple filaments that formed meiotic triple helices (MTHs) which are not present in interphase cells (Figure 1). It was also determined that MTHs are formed in pachytene I of yeast meiosis as synaptonemal complexes (SCs) began to appear (Ma et al., 2022).

Although nuclear actin bundles have been detected in meiotic yeast, there has been no evidence of these structures in the nuclei of somatic or meiotic cells in metazoans. However, under specific conditions such as DMSO treatment (Fukui and Katsumaru, 1980), overexpression (Kalendova et al., 2014; Baarlink et al., 2017), or by detection using an actin chromobody (Baarlink et al., 2013), bundled actin in the nuclei of somatic cells has been detected. Nevertheless, the monomeric state is the only form of actin for which there is some information where a parallel, in regards to function, between somatic and prophase I nuclei can be drawn. It has been shown that monomeric actin is an essential component of chromatin remodeling complexes in metazoans, however, since information of its role in chromatin associated events during prophase I was not the primary focus of previous investigations, we will discuss the involvement of actin in chromatin remodeling complexes in somatic nuclei and propose a model of actin involvement in the regulation of prophase I events.

## 4 Model of actin involvement in chromatin remodeling complexes in prophase I

Chromatin remodeling complexes execute changes in chromatin architecture that allow access of regulatory proteins to condensed genomic DNA (Saha et al., 2006; Magana-Acosta and Valadez-Graham, 2020). Since chromatin remodeling requires the coupling of several functions such as site-specific targeting, enzymatic and binding activity, and other modifying co-factors, proteins that are required for these functions are assembled into large multimeric chromatin remodeling complexes (Reyes et al., 2021). During meiosis, chromatin remodeling factors significantly accelerate nucleosome dynamics which promote rapid meiotic prophase I progression in an organized manner. The failure to undergo chromatin remodeling leads to a reduction in the effectivity of homologous pairing resulting in errors in recombination and germ-cell apoptosis (Prieto et al., 2005; Colas et al., 2008; Kota and Feil, 2010).

A unique feature of some of the chromosome remodeling complexes is the presence of monomeric actin, which is essential for their proper function. As has been described in somatic nuclei, monomeric actin in these complexes is always associated with Arp proteins (Kapoor and Shen, 2014; Klages-Mundt et al., 2018) or other ABPs (Zheng et al., 2009; Rajakyla and Vartiainen, 2014; Kristo et al., 2016) which together form a DNA binding module. In mammals, 11 Arp proteins have been described, four of which (Arp 1, 2, 3, and 10) have been shown to be cytoplasmic and are involved in actin filament nucleation and cytoskeleton regulation, while four others (Arp 4, 5, 6, and 8) are located in the nucleus (Ohfuchi et al., 2006; Aoyama et al., 2008; Kitayama et al., 2009). These nuclear Arps are recruited to chromatin remodeling complexes, including INO80, SWI/SNF and NuA4, via binding to a protein that contains a helicase-SANT-associated (HSA) domain, the sequence variations of which is specific for a particular Arp in the actin/Arp DNA binding module (Szerlong et al., 2003). In 2016, Cao et al. crystallised the actin/Arp module and showed that the actin inner face along with its barbed end is sequestered, which therefore masked its interaction with Arp4 and HSA. This masking prevents nuclear actin from polymerization as well as inhibits binding to actin regulators such as tropomodulin and thymosin-β4 or toxins latrunculin and phalloidin which target actin in the cytoplasm. Thus, this precludes regulation of nuclear actin by ATP hydrolysis which is required for polymerization features that together distinguish nuclear actin from its cytoplasmic counterpart (Cao et al., 2016).

In the following chapters, we will discuss the specific role of three chromatin remodeling complexes, INO80, SWI/SNF, and NuA4, all of which contain an actin-associated DNA-binding module. Importantly, since these complexes have been found not only in the nuclei of somatic cells but also in the nuclei of prophase I spermatocytes, we will propose how monomeric actin in these complexes may contribute to the regulation of meiotic progression. In addition, we will discuss our recent data pertaining to the Nucleosome Remodeling and Deacetylase (NuRD) complex which in addition to actin contain an ABP, vinculin, which seems to position the spermatoprotesome within the vicinity of chromatin remodeling complexes.

### 4.1 Actin recruits INO80 to DNA sites for active transcription and DNA break repair

The ATP-dependent chromatin remodeler complex, INO80, binds nucleosome-free regions around promoter and transcriptional start sites, organizes chromatin architecture by repositioning nucleosomes (Clapier and Cairns, 2009) and actively engages in DNA damage repair (Ebbert et al., 1999). INO80 consists of three ATPase subunits: the core ATPase subunit and two additional ATPase-containing helicases – RUVBL1 and RUVBL2, both of which play a role in the scaffolding of large protein assemblies (Chen et al., 2011). Within the INO80 complex, actin is associated with two Arp proteins, Arp4 and Arp8 (Oma and Harata, 2011; Tosi et al., 2013), a configuration that has been shown to be evolutionarily conserved from yeast to humans (Cai et al., 2007; Wu et al., 2007).

In somatic cells, actin facilitates the docking and movement of the INO80 complex at DNA sites and alters the composition of the nucleosome in an ATP-dependent manner with Arp4 and Arp8 binding to extranucleosomal DNA (Brahma et al., 2018). These Arps act as sensors along DNA by allosterically regulating INO80-mediated nucleosome spacing. Additionally, Arp8 is also recruited to DSB, initiating the repair process (Osakabe et al., 2014; Takahashi et al., 2017). Hence, both Arp4 and Arp8 are instrumental in the modulation and enhancement of nucleosome binding affinity. It is of note that Arp5 is localized at a distinct part of the complex and is essential for the coupling of ATP hydrolysis and nucleosome sliding (Yao et al., 2015; Yao et al., 2016). This arrangement was supported by Zhang et al., 2019 who recently provided the crystal structure of the INO80 complex which depicted the binding of the actin/Arp module to DNA, thus illustrating its presence independent of the ATPase subunit.

**FIGURE 2:**
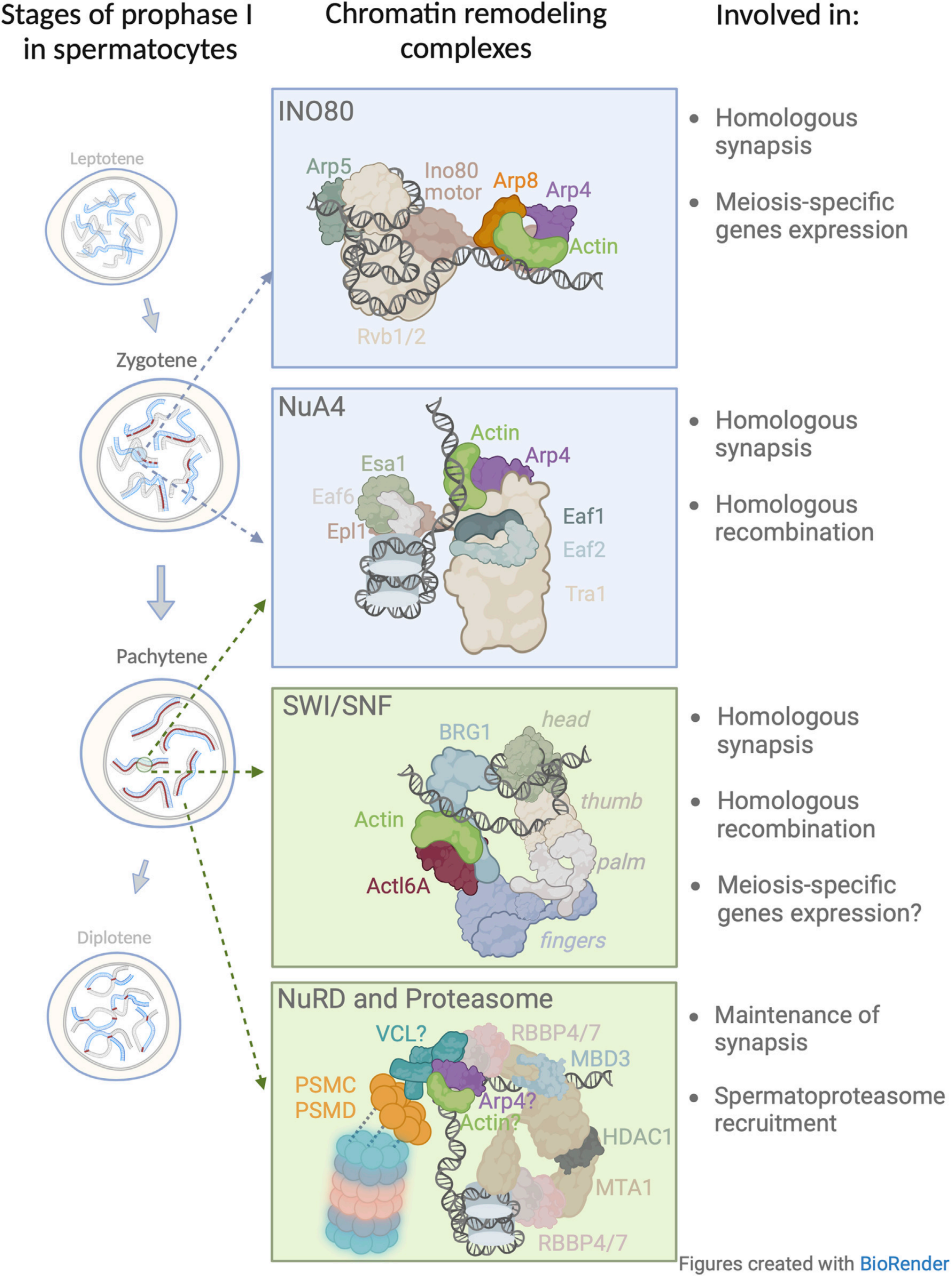
Model of nuclear actin involvement in chromatin remodeling during the prophase I. Four chromatin remodeling complexes in which actin has been implicated or proposed to be involved during prophase I. The presented structures and subunit nomenclature of these complexes were adapted from relevant studies: INO80 (Knoll et al., 2018); NuA4 (Qu et al., 2022); SWI/SNF (Wanior et al., 2021); NuRD (Arvindekar et al., 2022). In this figure, the model for NuRD complex in meiosis is based on a recent crystallographic study (Arvindekar et al., 2022) and extended to include newly identified subunits by Petrusova et al., 2022. Vinculin (VCL) associates with the 19 S cap of the spermatoproteasome (blue/pink barrel structure). The question mark which appears next to VCL, actin, and Arp4 depicts the possible presence of these subunits within the NuRD complex.

Analogous to DNA damage repair in somatic cells, during meiosis in both budding yeast and humans, INO80 is recruited to DSBs for effective DNA end resection and repair (Gospodinov et al., 2011) during homologous recombination (Osakabe et al., 2014). In the context of DSBs, Serber et al., 2016 set out to determine the role of INO80 during prophase I and discovered that male mice that had been conditionally depleted of INO80 exhibited impaired synaptonemal complex formation of prophase I spermatocytes, aberrant cross-over formation, and a diminished capacity to repair DSBs, resulting in sterility. In addition, Chakraborty and Magnuson, 2022 showed that INO80 is a negative regulator of poised chromatin, i.e., it is required for the regulation of spermatogenic gene expression. Since in somatic nuclei the actin/Arp module is essential for INO80 DNA binding, and at the same time INO80 is critical for prophase I, we propose that the actin/Arp module is important in prophase I chromatin remodeling (Figure 2).

### 4.2 Actin/Arp deposits NuA4 on chromatin loops for efficient homologous recombination

The NuA4 acetyltransferase complex in somatic nuclei is involved in chromatin compaction (Lu et al., 2009), transcriptional regulation (Lindstrom et al., 2006), and DSB repair (Bird et al., 2002; Downs et al., 2004; Lin et al., 2008; Torres-Machorro et al., 2015; Cheng et al., 2018). Recently, it has been shown that NuA4 regulates DNA damage repair by nudging homologous recombination to proceed over non-homologous end-joining in somatic nuclei (Harata et al., 1999; Galarneau et al., 2000; Torres-Machorro et al., 2015). This complex contains an actin/Arp module which helps NuA4 bind to DNA (Harata et al., 1999; Galarneau et al., 2000) and influences the docking of the catalytic domain, Esa1, to the nucleosome (Wang et al., 2018; Zukin et al., 2022).

Wang et al., 2021 showed that meiosis-specific depletion of Esa1 resulted in a decrease in chromosome axis length which consequently lead to a decrease in the number of DSBs, recombination intermediates, and crossover frequencies. However, this study did not examine the role of actin/Arp module in Esa1 loading on DNA, hence, it would be interesting to determine if in the absence of the actin/Arp module, there would be a phenotype similar to that observed in the Esa1 meiotic knock-out. Although this data shed light on the importance of NuA4 in prophase I progression (Figure 2), the exact role of the actin/Arp module in this complex remains to be established.

### 4.3 Actin in SWI/SNF affects chromatin reorganization

The mammalian SWI/SNF complex, also known as BAF (Hodges et al., 2016), is a macromolecular assembly that functions as a gene regulator, influencing changes in chromatin structure and accessibility (Peterson et al., 1998; Zhao et al., 1998). It is comprised of either the ATP-ase subunit catalytic subunit BRG1 or BRM along with core subunits, including β-actin (Hodges et al., 2016; Wang et al., 2018; Wang et al., 2021). Importantly, it is the β-actin isoform that maximizes the ATPase activity of BRG1 (Peterson et al., 1998), and in turn, promotes chromatin remodeling activity, nucleosome compaction, and transcriptional regulation (Morris et al., 2014). Mechanistically, Xie et al., 2018 determined that in the absence of β-actin, BRG1 is disassociated from chromatin resulting in the reorganization of chromatin and alteration of the expression of genes involved in angiogenesis and cytoskeletal organization which lead to changes in cellular identity. It should be taken into consideration that the cells used in this study were derived from mouse embryonic fibroblasts (MEFs) obtained from a β-actin knockout mouse model that lacked both cytoplasmic and nuclear actin. The authors provided evidence which demonstrated that the reintroduction of nuclear-targeted actin into these MEFs restored some nuclear features and gene expression. From this result, they concluded that nuclear β-actin can affect the genome-wide organization of heterochromatin, through BRG1 heterochromatin binding activity (Xie et al., 2018). Building on this result, Mahmood et al., 2021 provided a clear link between nuclear β-actin, chromatin accessibility, and compartment-level changes in genome organization in the same MEFs. Using advanced technologies such as ATAC-Seq, HiC-Seq, RNA-Seq, and ChiP-Seq, the authors showed that the loss of nuclear β-actin induced changes in the 3D architecture of heterochromatin and euchromatin compartments, and hence influenced the regulation of genes involved in development and differentiation (Mahmood et al., 2021). These findings provided evidence of the crucial role of nuclear β-actin in the regulation of chromosomal structure.

In mouse germ cells, Wang et al., 2012 showed that the deletion of BRG1 impaired homologous recombination causing spermatogenesis arrest at the mid-pachytene stage, ultimately leading to apoptosis. More specifically, BRG1*-*depleted spermatocytes showed dysregulated gene expression patterns and altered chromatin organization. Additionally, Menon et al., 2019 confirmed that in spermatocytes, BRG1 upregulated the genes involved in the progression of meiosis and spermatogonial pluripotency, while genes which were not involved in the meiotic process were generally downregulated.

To the best of our knowledge, the role of β-actin in prophase I events has not been addressed. Assuming that the role and composition of SWI/SNF in somatic cells is similar in germ cells, then comprehending the role of β-actin in prophase I would be of great interest. In regards to the mechanisms of SWI/SNF that take place in somatic and meiotic cells, we posit that β-actin partners with BGR1, and thus represents a vital part of chromatin organization during mid-prophase I (Figure 2), affecting chromatin condensation, gene expression, and homologous recombination.

### 4.4 Vinculin, actin and Arp4—potential “new kids on the block” within the NuRD complex

As previously mentioned, there are more than 30 ABPs that localize to the nucleus. We recently showed that one such ABP, vinculin, resides in the nuclei of primary spermatocytes (Petrusova et al., 2022). So far, vinculin activity has only been studied in the cytoplasm where it is known to localize to focal adhesions in adherent somatic cells and facilitates mechanotransduction in cell-matrix and cell-cell adhesions by transmitting forces between the cytoskeleton, extracellular matrix as well as cell-to-cell connections (Goldmann, 2016; Bays and DeMali, 2017). Vinculin forms a trestle-like structure as a result of its interaction with approximately 20 other molecules to connect the cell membrane with the actin cytoskeleton (Jannie et al., 2015). Importantly, it orchestrates the polymerization of the actin cytoskeleton at the proximity of the plasma membrane (Bays and DeMali, 2017) and crosslinks actin filaments to form actin bundles (Janssen et al., 2006; Shen et al., 2011).

We have recently shown that vinculin is an essential element for prophase I progression in male mice (Petrusova et al., 2022). Vinculin accumulates on newly formed homologous chromosome pairs in both zygotene and pachytene stages, and interestingly, has been found in greater concentrations on the centromeres of chromosome tetrads during the pachytene stage. To better understand the involvement of nuclear vinculin in prophase I events, we utilized mass spectrometry (MS) to determine if notable binding partners could be detected during prophase I. This analysis yielded 38 candidates, two of which, RBBP4 and RBBP7, have been shown to be components of the NuRD chromatin associated complex (Xue et al., 1998; Bode et al., 2016; Bornelov et al., 2018). It is of note, that when the NuRD complex was functionally disabled in primary spermatocytes, erroneous synapsis formation, premature chromosomal splitting, and metaphase I arrest were observed (de Castro et al., 2022). Remarkably, the same phenotype was identified when vinculin was conditionally ablated in primary spermatocytes (Petrusova et al., 2022). Therefore, these overlapping phenotypes may serve as a testament of the coupling between NuRD and vinculin, the combination of which may have the common purpose of regulating specific events of prophase I.

Unexpectedly, we found a number of regulatory subunits including PSMC (1 through 6) and PSMD (Humphries et al., 2007; Plessner et al., 2015; Kristo et al., 2016; Lappalainen, 2016; Henderson et al., 2017; Virtanen and Vartiainen, 2017; Virtanen and Vartiainen, 2017; Caridi et al., 2018) of the 19S proteasome cap among vinculin’s interacting partners (Petrusova et al., 2022). Interestingly, proteasomes, in general, are readily detected in prophase I nuclei and found in proximity to chromosomes where they play a pivotal role in the regulation of meiosis (Ahuja et al., 2017; Rao et al., 2017; Vujin and Zetka, 2017), notably in chromatin remodeling, transcriptional regulation, and DNA damage repair (McCann and Tansey, 2014; Zou and Mallampalli, 2014; Vaughan et al., 2021; Guo, 2022). The coupling of prophase I events with proteasome function has been shown to be evolutionarily conserved in organisms such as *S. cerevisiae*, *C. elegans*, and the mouse. Importantly, the proteasome in the nuclei of spermatocytes, i.e., spermatoproteasome complex, differs from other mammalian proteasomes in its catalytic 20 S core subunit mostly due to the enrichment of its PA200 subunit, which is responsible for binding and subsequent degradation of acetylated core histones (Qian et al., 2013). This is likely important for the regulation of and the continuity from homologous chromosome pairing to the generation of homologous recombination intermediates (Brown et al., 2008; Bhagwat et al., 2021) and segregation-competent cross-overs (Ahuja et al., 2017; Rao et al., 2017). In vinculin depleted spermatocytes, the spermatoproteasome loses its ability to localize to chromosomes and disappears from the nucleoplasm (Petrusova et al., 2022). Therefore, it appears that vinculin may not be only involved in meiotic chromatin remodeling processes but also in the positioning of degradation machinery at meiotic chromosomes to remove proteins that are no longer required. This may allow the recruitment of other factors needed for the coordinated and successive sequence of prophase I events. Interestingly, along with other NuRD partnering subunits, we also detected the presence of actin and Arp4 with vinculin. Since the actin/Arp4 module in other chromatin remodeling complexes, mediates the interaction with DNA, this same function could be assumed for this module in NuRD complex. Understanding the intricacies of vinculin and the actin/Arp4 module may provide an explanation for the binding of the NuRD complex to DNA (Figure 2).

## 5 Conclusion and future perspectives

Chromatin remodeling complexes are essential regulators that exhibit a broad range of functions in cellular processes such as transcription and DNA metabolism. While the function of these complexes was initially studied in established cell lines, their role in prophase I of meiosis have only been recently interrogated. Data from various mouse models has been particularly informative, since they have allowed researchers to decipher the function of some of the components of each remodeling complex, and have also provided a window into their role in fertility. This not only implicates nuclear actin as an essential part of chromatin-remodeling complexes in somatic cell nuclei but also in prophase I of meiosis. In addition, at least in the case of the NURD complex, we believe that chromatin remodeling complexes coupled via vinculin to spermatoproteasomes may represent a mechanism that is necessary allowing a highly ordered sequence of events of Prophsae I to proceed quickly and efficiently. Should this coupling be found to be a common feature for other chromatin remodeling complexes which participate in Prophase I, it may provide the experimental framework to study whether the processivity of Prophase I is regulated in the context of local protein degradation. While the data generated over the past few years has been intriguing, more physiologically relevant experiments are needed to garner a full understanding of the role of these complexes in gametogenesis.

The presence of nuclear actin in chromatin remodeling complexes has been shown by MS analysis and crystallography. Unfortunately, gene-specific ablation approaches are hampered by the fact that nuclear monomeric actin appears to be ubiquitous in chromatin remodeling complexes. Thus, the optimum method to determine the specific role of actin in each complex would be to mutate the sequences of neigboring subunits with which actin interacts. However, this would certainly require an understanding of the steric details of these interactions, given the uniqueness of each complex. Additionally, to confirm the presence and interaction of actin, vinculin, and other ABPs with these complexes in prophase I of meiosis, it would be beneficial to generate high-resolution microscopic images. The only reported image of nuclear actin relates to its appearance as submicron length nuclear structures, however these structures have not observed in or around chromatin-rich regions. This is likely explained by the fact that actin epitopes that are recognized by relevant probes that detect actin are inaccessible as they are located within the chromatin remodeling complex or masked by other components, therefore, a microscopic approach may not be optimal. To obtain a clearer picture of this interaction, the development of new molecular probes or other high-performance molecular methods, such as cryo-electron microscopy, could be instrumental. In summary, understanding the role of nuclear actin as a common denominator in chromatin remodeling complexes will provide clarity of the structural and functional plasticity in meiosis as well as in the cell cycle in general.
